# HTR3A and HTR3E gene polymorphisms and diarrhea predominant irritable bowel syndrome risk: evidence from a meta-analysis

**DOI:** 10.18632/oncotarget.19682

**Published:** 2017-07-29

**Authors:** Tangming Guan, Ting Li, Weiming Cai, Dong Huang, Peipei Ouyang, Yan Wang, Huayan Chen, Kefeng Wu, Xiaoli Ma

**Affiliations:** ^1^ Department of Pharmacy, Affiliated Hospital of Guangdong Medical University, Zhanjiang, Guangdong Province 524001, China; ^2^ Laboratory of Clinical Pharmacy, Guangdong Medical University, Zhanjiang, Guangdong Province 524001, China; ^3^ Department of Pharmacology, Guangdong Medical University, Zhanjiang, Guangdong Province 524001, China; ^4^ Guangdong Key Laboratory for Research and Development of Natural Drug, Guangdong Medical University, Zhanjiang, Guangdong Province 524001, China; ^5^ Department of Clinical Pharmacy, Guangdong Medical University, Zhanjiang, Guangdong Province 524001, China

**Keywords:** HTR3A, HTR3E, polymorphism, meta-analysis, diarrhea predominant irritable bowel syndrome

## Abstract

Several studies have reported an association between serotonin receptor type 3 (5-HT_3_) subunit genes *HTR3A* (rs1062613) and *HTR3E* (rs62625044) and diarrhea predominant irritable bowel syndrome (IBS-D). However, the results remain inconclusive and controversial, particularly for the data derived from different ethnicities and genders. Therefore, we performed a meta-analysis to evaluate this association. All eligible case-control studies that met the search criteria were retrieved from multiple databases, and five case-control studies were included for detailed evaluation. The pooled odds ratios (ORs) with 95% confidence intervals (95% CIs) were calculated to assess the strengths of the associations of *HTR3A* (rs1062613) and *HTR3E* (rs62625044) polymorphisms with IBS-D risk. Our results revealed statistically significant associations of the *HTR3A* (rs1062613, C/T) polymorphism with a decreased risk of IBS-D in all genetic models. Additionally, the HTR3E (rs62625044, G/A) polymorphism was also found to be significantly associated with a decreased risk of IBS-D in the allele and recessive models. Subgroup analysis revealed that these associations held true especially for Asians and female. In conclusion, this meta-analysis suggested that the C allele of *HTR3A* (rs1062613) and the G allele of *HTR3E* (rs62625044) are associated with a decreased risk of IBS-D.

## INTRODUCTION

Irritable bowel syndrome (IBS) is one of the most common gastrointestinal disorder characterized by abdominal discomfort, pain, and altered bowel habits; it may considerably reduce patients’ quality of life and work productivity, which affects more than 7 percent of people all around the world [1, 2]. According to the recurrent symptoms, IBS patients can experience constipation (IBS-C), diarrhea (IBS-D), or both (IBS-M) [3, 4]. IBS is a multi-factorial disorder disease associated with biological and psychosocial factors [5–9]. However, the etiology of IBS remains largely unknown. Genetic predisposition has been demonstrated in classical family/twin studies and epidemiological surveys, but unequivocal susceptibility genes have yet to be identified [2, 10]. Recently, several genetic association studies identified the serotonin receptor type 3 (5-HT_3_) subunit genes *HTR3A* and *HTR3E* polymorphisms as being significantly associated with IBS ( particularly IBS-D) [11–14].

The 5-HT_3_ receptor is a Cys-loop ligand-gated ion channel composed of five subunits [15], which are encoded by the *HTR3A, HTR3B*, *HTR3C, HTR3D* and *HTR3E* genes [16–18]. Different *HTR3* variants have been reported to be associated with schizophrenia [19], depression [20], anxiety [21], autism [22], obsessive compulsive disorder (OCD) [23], drug abuse and addiction [24, 25]. Recently, the associations between these two *HTR3* polymorphisms (*HTR3A* and *HTR3E*) and the risk of IBS-D have been intensively investigated. However, the community is still unable to reach a consensus, particularly regarding the data from different ethnicities and genders [11–14, 26]. Such inconsistencies are generally due to ethnic differences or variations in sample size. Hence, we designed this meta-analysis to quantify the overall genetic effects of the *HTR3A* and *HTR3E* polymorphisms on the risk of IBS-D.

## RESULTS

### Characteristics of studies

**Figure 1:**
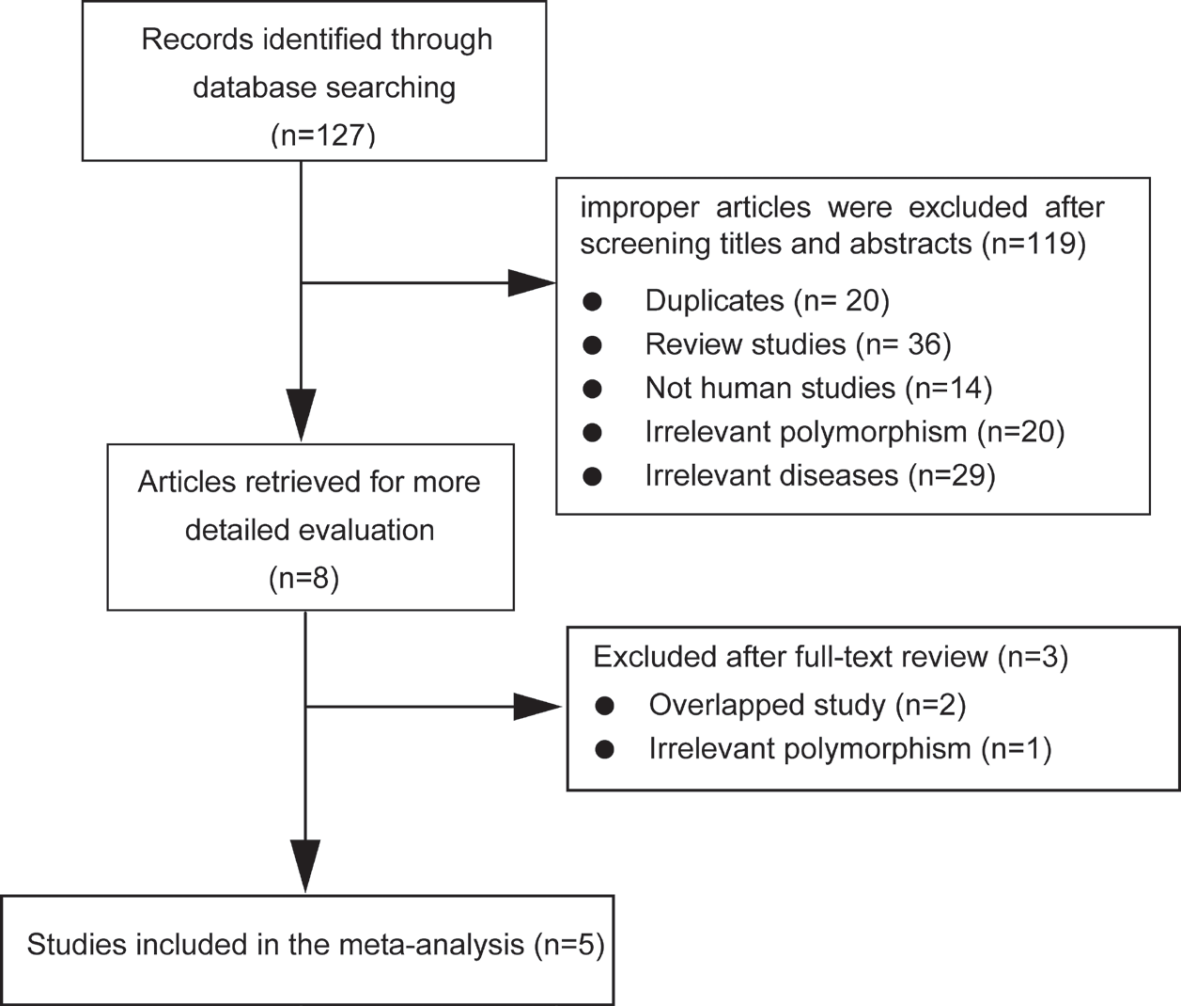
Flow diagram of selection of eligible studies

As showed in Figure 1, 5 studies involving 1,287 cases and 1,418 controls were ultimately included in the present meta-analysis. Regarding the *HTR3A (*rs1062613, C/T) polymorphism, 5 studies were available and included a total of 1,178 cases and 1,124 controls. Regarding the *HTR3E (*rs62625044, G/A) polymorphism, 5 studies involving a total of 1,276 cases and 1,389 controls were available. All of these included studies were hospital-based, of which 3 from China, 1 from Germany, 1 from USA. Additionally, all of the included studies were of high quality, as indicated by the Newcastle-Ottawa scale (NOS) scores of each study being above 6 points (see Supplementary Table 1), and the genotype distributions in all of the controls were consistent with Hardy-Weinberg equilibrium (HWE), except one [6]. Studies with controls not in HWE were also considered for the meta-analysis, but they were excluded in the sensitivity analysis. The main characteristics of the included studies were summarized in Table 1.

**Figure 2:**
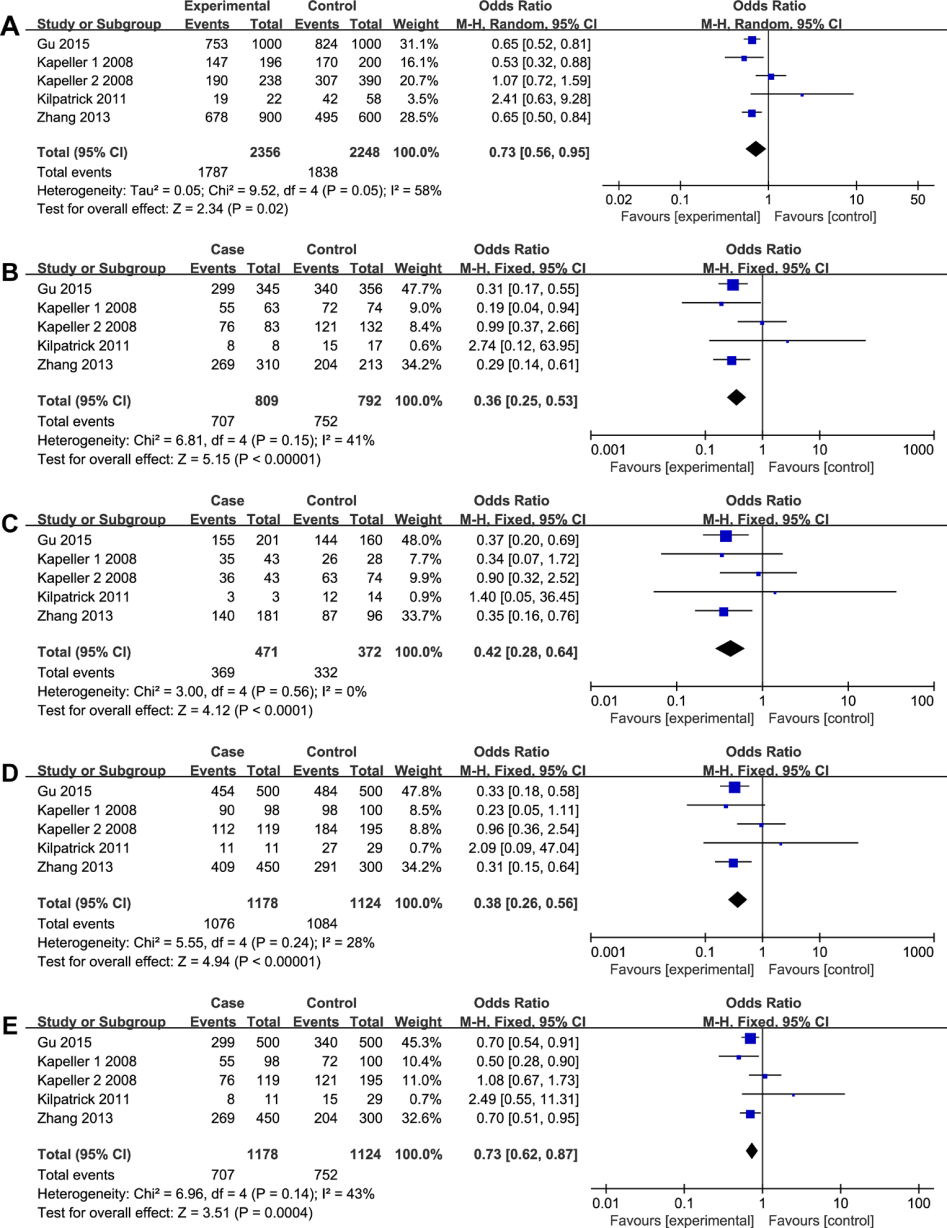
The associations of *HTR3A (*rs1062613, C/T) with ISB-D in different genetic models (**A**) Allele model (C vs. T). (**B**) Codominant model (CC vs. TT). (**C**) Codominant model (CT vs. TT). (**D**) Dominant model (CC + CT vs. TT). (**E**) Recessive model (CC vs. TT + CT).

**Figure 3:**
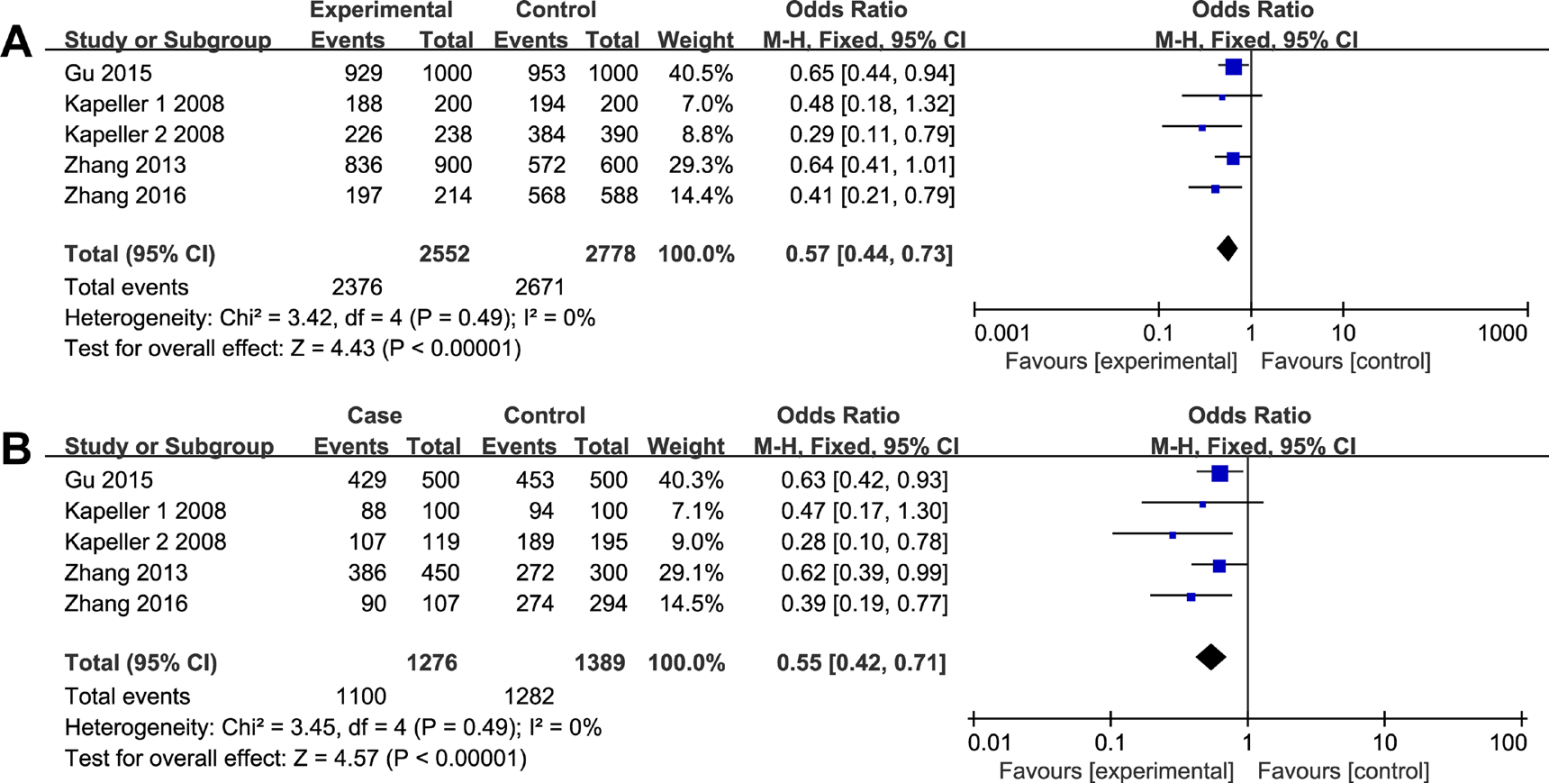
The associations of *HTR3E (*rs62625044, G/A) with ISB-D in different genetic models (**A**) Allele model (G vs. A). (**B**) Recessive model (GG vs. GA+AA).

**Figure 4:**
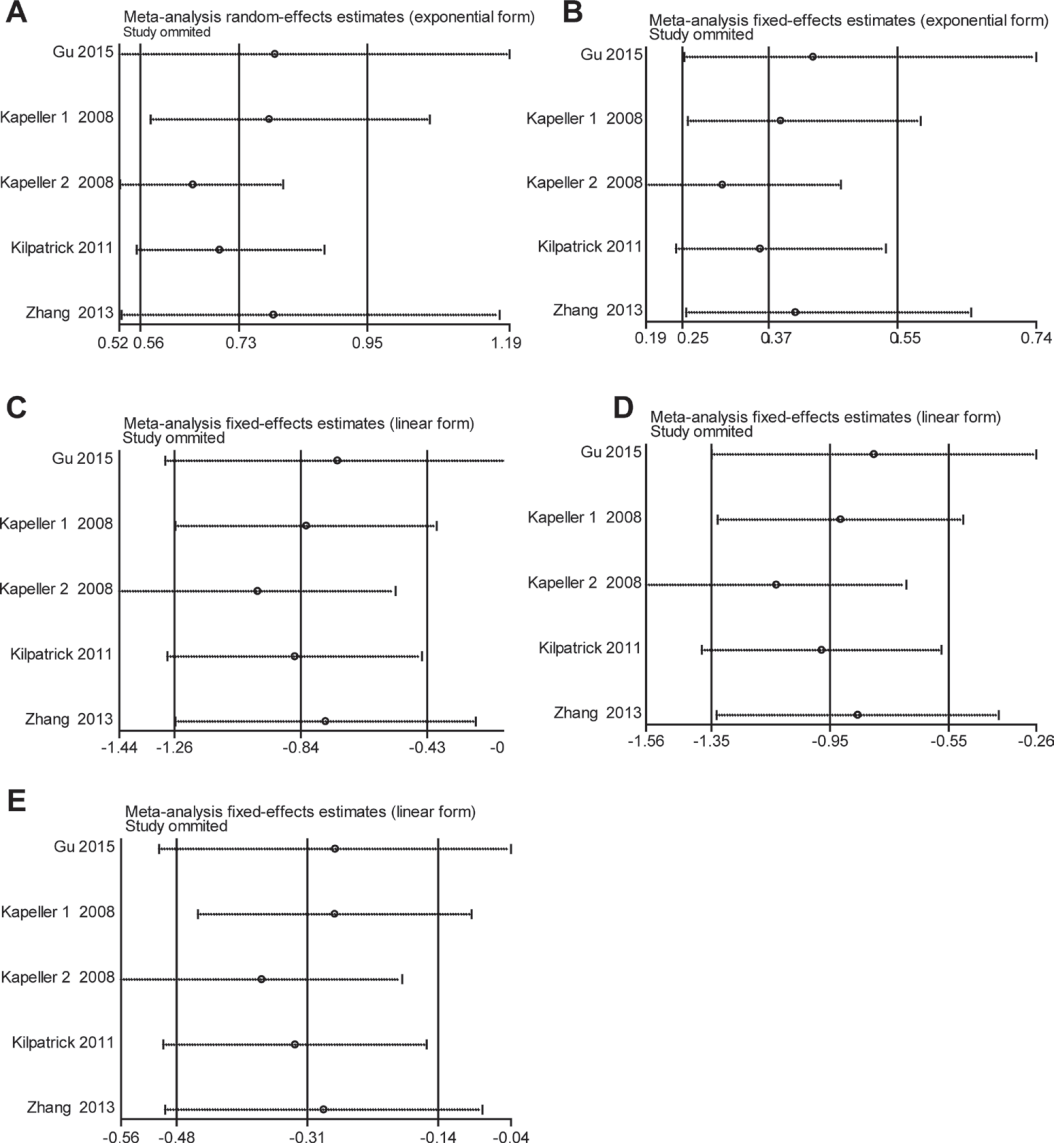
Sensitivity analysis of the association of *HTR3A (*rs1062613, C/T) and ISB-D in the different genetic models (**A**) Allele model (C vs. T). (**B**) Codominant model (CC vs. TT). (**C**) Codominant model (CT vs. TT). (**D**) Dominant model (CC + CT vs. TT). (**E**) Recessive model (CC vs. TT + CT).

**Figure 5:**
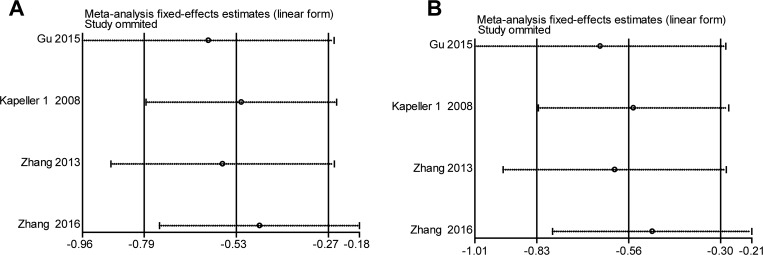
Sensitivity analysis of the association of *HTR3E (*rs62625044, G/A) and ISB-D in the different genetic models (**A**) Allele model (G vs. A). (**B**) Recessive model (GG vs. AA + AG).

**Table 1:** Characteristics of studies included in the meta-analysis

Study	Country	Ethnicity	Design	HTR3A (case/control)			HTR3E (case/control)			Genotyping Method	Diagnosis criteria	HWE (P)		NOS
				CC	CT	TT	GG	GA	AA			HTR3A	HTR3E	
Gu 2015	China	Asian	HB	299/ 340	155/ 114	46/ 16	429/ 453	71/ 47	0/ 0	PCR and RFLP	Rome II	0.87	0.27	6
Kapeller 1 2008	Germany	Caucasian (UK)	HB	55/ 72	35/ 26	8/ 2	88/ 94	12/ 6	0/ 0	PCR and sequencing	Rome II	0.84	0.76	7
Kapeller 2 2008	Germany	Caucasian, (Germany)	HB	76/ 121	36/ 63	7/ 11	107/ 189	12/ 5	0/ 1	PCR and sequencing	Rome II	0.46	0.00	7
Kilpatrick 2011	USA	American	HB	8/ 15	3/ 12	0/ 2	-	-	-	TaqMan	Rome II	0.85	-	7
Zhang 2013	China	Asian	HB	269/ 204	140/ 87	41/ 9	386/ 272	64/ 28	0/ 0	PCR and RFLP	Rome III	0.64	0.59	6
Zhang 2016	China	Asian	HB	-	-	-	90/ 274	17/ 20	0/ 0	PCR and RFLP	Rome III	-	0.55	7

Abbreviations: HB, hospital-based study; PB, population based; Uk, United Kingdom.

RFLP, restriction fragment length polymorphism analyses.

### Power analysis

Before implementation of this meta-analysis, statistical power was assessed with the assumptions:α err prob = 0.05, OR = 1.30 (corresponding to a “weak to moderate” gene effect) for the two single nucleotide polymorphisms (SNPs), and minor allele frequencies (MAF) of *HTR3A* (rs1062613, C/T) and *HTR3E* (rs62625044, G/A) were estimated from the 1000 Genomes. The present samples indicated that 97.8% power for *HTR3A* (rs1062613, C/T) (MAF = 0.25), and 17.5% power for *HTR3E* (rs62625044, G/A) (MAF = 0.01). The power analysis indicated that these recruited samples could provide sufficient power in identifying the association between *HTR3A (*rs1062613, C/T) polymorphism and IBS-D.

### Quantitative synthesis

As illustrated in Figures 2–3, different genetic models of *HTR3A (*rs1062613, C/T) and *HTR3E (*rs62625044, G/A) were used in our analysis.

The C allele of rs1062613 was found to be significantly associated with a decreased risk of IBS-D. The following data were obtained: in the allele model, C vs. T, OR = 0.73, 95% CI: 0.56–0.95, and *P* = 0.02; in the codominant model, CC vs. TT, OR = 0.36, 95% CI: 0.25–0.53, *P* < 0.00001; CT vs. TT, OR = 0.42, 95% CI: 0.28–0.64, and *P* < 0.0001; in the dominant model, CC+CT vs. TT, OR = 0.38, 95% CI: 0.26–0.56 and *P* < 0.0001; and recessive (CC vs. CT+TT, OR = 0.73, 95% CI: 0.62–0.87, *P* = 0.0004) (Figure 2). In the subgroup analyses by ethnicity, the significant association was found among Asians (C vs. T, OR = 0.65, 95% CI: 0.55–0.77, *P* < 0.00001; CC vs. TT, OR = 0.30, 95% CI: 0.19–0.47, *P* < 0.00001; CT vs. TT, OR = 0.37, 95% CI: 0.23–0.59, *P* < 0.0001; CC+CT vs. TT, OR = 0.32, 95% CI: 0.20–0.50, *P* < 0.00001; CC vs. CT+TT, OR = 0.70, 95% CI: 0.57–0.85, *P* = 0.0004 ), while no significant association was found among non-Asians. In addition, analysis stratified by different gender showed a significant association of IBS risk with the C allele deletion in both female and male. All the results are listed in Table 2.

**Table 2:** Summary of meta-analysis for the association of *HTR3E* (rs62625044, G/A) and *HTR3E* (rs62625044, G/A) polymorphisms with ISB-D

Genetic model	Stratifications	Number of studies	OR (95%CI)	*P* value	Heterogeneity	
					*I*^2^	*P*_H_
**HTR3A (rs1062613, C/T)**						
**C vs. T**	Overall	5	0.73 [0.56, 0.95]	0.02	58%	0.05
	Asians	2	0.65 [0.55, 0.77]	< 0.00001	0%	0.98
	Non-Asians	3	0.93 [0.47, 1.82]	0.82	71%	0.03
	female	5	0.71 [0.60, 0.85]]	0.0001	32%	0.21
	male	4	0.61 [0.46, 0.81]	0.0006	30%	0.24
**CC vs. TT**	Overall	5	0.36 [0.25, 0.53]	< 0.00001	41%	0.15
	Asians	2	0.30 [0.19, 0.47]	< 0.00001	0%	0.91
	Non-Asians	3	0.65 [0.31, 1.37]	0.26	47%	0.26
	female	5	0.40 [0.26, 0.61]	< 0.0001	0%	0.41
	male	4	0.20 [0.07, 0.57]	0.002	27%	0.25
**CT vs. TT**	Overall	5	0.42 [0.28, 0.64]	< 0.0001	0%	0.56
	Asians	2	0.37 [0.23, 0.59]	< 0.0001	0%	0.91
	Non-Asians	3	0.69 [0.31, 1.54]	0.36	0%	0.56
	female	5	0.46 [0.30, 0.72]	0.0006	0%	0.77
	male	4	0.25 [0.08, 0.73]	0.01	0%	0.43
**CC+CT vs. TT**	Overall	5	0.38 [0.26, 0.56]	< 0.00001	28%	0.24
	Asians	2	0.32 [0.20, 0.50]	< 0.00001	0%	0.91
	Non-Asians	3	0.66 [0.31, 1.39]	0.27	29%	0.25
	female	5	0.42 [0.28, 0.63]	< 0.0001	0%	0.52
	male	4	0.22 [0.08, 0.60]	0.003	21%	0.29
**CC vs. CT+TT**	Overall	5	0.73 [0.62, 0.87]	0.0004	43%	0.14
	Asians	2	0.70 [0.57, 0.85]	0.0004	0%	1.00
	Non-Asians	3	0.91 [0.44, 1.89]	0.80	67%	0.05
	female	5	0.75 [0.61, 0.93]	0.008	0%	0.40
	male	4	0.66 [0.48, 0.90]	0.009	5%	0.37
**HTR3E (rs62625044, G/A)**						
**G vs. A**	Overall	5	0.57 [0.44, 0.73]	< 0.00001	0%	0.49
	Asians	3	0.60 [0.46, 0.79]	0.0002	0%	0.47
	Non-Asians	2	0.38 [0.19, 0.77]	0.007	0%	0.49
	female	5	0.41 [0.30, 0.56]	< 0.00001	0%	0.59
	male	4	1.09 [0.71, 1.68]	0.69	0%	0.97
**GG vs. GA+AA**	Overall	5	0.55 [0.42, 0.71]	< 0.00001	0%	0.49
	Asians	3	0.58 [0.44, 0.77]	0.0001	0%	0.46
	Non-Asians	2	0.36 [0.18, 0.75]	0.006	0%	0.49
	female	5	0.39 [0.28, 0.54]	< 0.00001	0%	0.59
	male	4	1.10 [0.70, 1.72]	0.68	0%	0.97

The G allele of rs62625044 was found to be significantly associated with a decreased risk of ISB-D in allele model (G vs. A, OR = 0.57, 95% CI: 0.44–0.73, *P* < 0.00001) and recessive model (GG vs. GA+AA, OR = 0.55, 95% CI: 0.42–0.71, *P* < 0.00001) (Figure 3). In the subgroup analyses by ethnicity, the significant association was found both in Asians (G vs. A, OR = 0.60, 95% CI: 0.46–0.79, *P* = 0.0002; GG vs. GA+AA, OR = 0.58, 95% CI: 0.44–0.77, *P* = 0.0001) and non-Asians (G vs. A, OR = 0.38, 95% CI: 0.19–0.77, *P* = 0.007; GG vs. GA+AA, OR = 0.36, 95% CI: 0.18–0.75, *P* = 0.006). In addition, analysis stratified by different gender showed a significant association of IBS-D risk with the G allele deletion in female(G vs. A, OR = 0.41, 95% CI: 0.30–0.56, *P* < 0.00001; GG vs. GA+AA, OR = 0.39, 95% CI: 0.28–0.54, *P* < 0.00001), but not in male. All the results are listed in Table 2.

### Sensitivity analysis

Sensitivity analysis of the summary odds ratio coefficients on the relationships of the two SNPs and the risk of IBS-D is computed by omitting each study in turn. The corresponding pooled ORs were not significantly altered after excluding each eligible study at a time (Figures 4–5).

### Publication bias

No evidence of publication bias was detected regarding the ORs of the two SNPs in this study by either Begg’s or Egger’s test (Table 3).

**Table 3:** Publication bias tests for association of the *HTR3A* (rs1062613, C/T) and *HTR3E* (rs62625044, G/A) polymorphisms with ISB-D

Comparisons	Egger test			Begg test
	Coefficient	*P* value	95% CI	*P* value
**HTR3A (rs1062613, C/T)**				
**C vs. T**	1.81	0.30	(−2.78, 6.41 )	0.81
**CC vs. TT**	1.20	0.44	(−3.12, 5.53 )	0.81
**CT vs. TT**	0.88	0.39	(−1.91, 3.68 )	0.22
**CC+CT vs. TT**	1.09	0.44	(−2.81, 4.99 )	0.22
**CC vs. CT+TT**	1.32	0.40	(−2.98, 5.61 )	0.81
**HTR3E (rs62625044, G/A)**				
**G vs. A**	−1.53	0.23	(−5.38, 2.31 )	0.31
**GG vs. GA+AA**	−1.56	0.23	(−5.51, 2.39 )	0.31

## DISCUSSION

Recently, several genetic association studies identified a novel association between *HTR3* (*HTR3A* and *HTR3E*) and IBS-D. In Europe, Kapeller J [11] found that the novel *HTR3E* variant c.*76G>A (rs62625044) is significantly associated with female IBS-D in two independent cohorts from the UK and Germany, while the *HTR3A* variant c.-42C>T (rs1062613) is only associated with the risk for IBS-D in UK, but not in Germany population. In Asia, Gu et al. [13] demonstrated that genetic polymorphisms in *HTR3A* and *HTR3E* are associated with the risk for D-IBS in Chinese population, especially in women. However, another studies [26] revealed that the *HTR3A* polymorphism loci rs1062613 may be not associated with IBS risk in American population. Due to relatively small samples from different populations, these studies demonstrated inconsistent results. Therefore, we performed a meta-analysis to estimate the association between *HTR3A* and *HTR3E* polymorphisms and the risk for IBS-D. To the best of our knowledge, this is the first meta-analysis to explore the relationships between *HTR3A* and *HTR3E* gene polymorphisms and IBS-D susceptibility. Five case-control studies [11–14, 26] with a total of 1,287 IBS-D patients and 1,418 healthy controls were included in our meta-analysis, which was giving a greater power to detect IBS-D risk associated with *HTR3A* and *HTR3E gene* polymorphisms.

In our meta-analysis, the main finding was that the C allele of *HTR3A (*rs1062613) decreases the risk of IBS-D in all of the comparison models, and the G allele of *HTR3E* (rs62625044) was also observed to have lower IBS-D risk in the allele and recessive models. Besides that, subgroup analyses by ethnicity indicated that SNP of rs62625044 (G/A) was significantly associated with a decreased risk of IBS-D in both Asian and non-Asian population, while the SNP of rs1062613(C/T) was only associated with the risk for IBS-D in Asian population. Moreover, analyses stratified by different gender showed a significant association between rs1062613(C/T) polymorphism and risk of IBS-D in both female and male population, while the SNP of rs62625044 (G/A) was only associated with risk of IBS-D in female population. Our results have some differences from the previous studies [11, 26]. A possible explanation for this phenomenon is that the previous single studies of IBS-D had small samples size, and thus the significance of current work may not be justified; thus, further studies are needed to clarify the effects of the 2 SNPs on the risk of IBS-D. Furthermore, the differential allele frequencies of the 2 SNPs exerted disproportionate levels of influence on the IBS-D risks in different populations. For example, the minor allele frequencies (MAFs) of the 2 SNPs rs1062613 and rs62625044 are 0.147 and 0.000, respectively, in the East Asian population (EAS), whereas the MAFs are 0.169 and 0.019, respectively, in the Ad Mixed American population (AMR) and the MAFs are 0.218 and 0.028, respectively, in the European population (EUR) based on the data from the 1000 G. Finally, significant heterogeneity was observed in allele model of rs62625044, and factors, such as ethnicity, gender distribution, psychosocial, genotyping method and other, might be potential sources of heterogeneity.

Additionally, the genotype distributions in all of the controls were consistent with HWE, except one for one study reported by Kapeller et al. [11]. However, the association was not significant change when excluded the study. The NOS results indicated that the included studies were credible. Moreover, sensitivity analysis was conducted, and it did not significantly alter the combined ORs. Additionally, no evidence of publication bias was identified by either Begg’s or Egger’s tests. Taken together, the results of this meta-analysis are reliable and stable.

There were some limitations in the current study. Firstly, the sample sizes of the studies included in our meta-analysis were relatively small, especially in the subgroup of non-Asians, which may lead to a false negative result. Therefore, the negative results of the non-Asians should be interpreted with caution. Moreover, although meta-analysis has the benefit to overcome this limitation and may generate more precise results, the combined sample sizes in our meta-analysis were still inadequate to detect the association between rs62625044 polymorphism and IBS-D risk because power calculation for the pooled sample sizes was less than 80%, which might attenuate the statistical power to detect a slight effect and increase the chance of opportunity bias. Second, due to the limited data, we did not carry out subgroup analysis to other factors, which may participate in the progression of IBS-D, such as age, lifestyle and social psychology. Thirdly, only articles in English and Chinese language were included; thus, studies written in other languages were neglected. Finally, although we performed a systematic searching strategy to identify eligible studies, there was still probability that few studies so called “grey literatures” were not included.

In conclusion, this meta-analysis suggested that the C allele of *HTR3A (*rs1062613) and the G allele of *HTR3E* (rs62625044) are associated with a decreased risk of IBS-D. One evidence indicates that these HTR3 polymorphisms can remarkably up-regulate the expression of 5-HT_3_ receptors, which have been proved to cause the gastric functional disorders including emesis, eating disorders and IBS-D, *et al.* [27], as illustrated in Supplementary Figure 1. Due to the above-mentioned limitations, a well-designed large-scale study that includes ethnicities, genders and psychosocial factors is required to confirm the findings of the current meta-analysis.

## MATERIALS AND METHODS

### Search strategy

In accordance with the Preferred Reporting Items for Systematic Reviews and Meta-Analyses (PRISMA) statement [28], we searched the related literature of the electronic records of the PubMed, Science Direct, Embase, WANFANG databases and Chinese National Knowledge Infrastructure (CNKI) published through 30 March 2017. The search terms included the following key words: (“Irritable bowel syndrome” *or* “IBS”) AND (“polymorphism” *or* “allele” *or* “genetic” *or* “gene” *or* “mutation” *or* “variant”) AND (“serotonin type 3” or “serotonin receptor 3” or “5-HT_3_” or *“HTR3A” or “HTR3E”)*. Furthermore, the references of all retrieved articles were also checked by hand to identify additional potential studies. The languages were limited to English and Chinese.

### Inclusion criteria

The inclusion criteria were as follows: (1) studies of the association between *HTR3A or HTR3E* polymorphism and IBS; (2) cohort or case-control study (3) provided sufficient data of allele and genotype frequencies of SNPs or required information could be calculated; and (4) if serial studies on the same population were published, only the most recent study was included. Additionally, we excluded reviews, abstracts, and redundant and animal studies.

### Data extraction

Two independent investigators extracted relevant data from all included studies on the basis of the inclusion criteria, and a third investigator verified them. The following information from eligible studies was extracted: the first author^’^s name, publication year, country of origin, ethnicity, sample size, genotyping method, and the *HTR3A and HTR3E* genotype distributions and alleles in the case and control groups.

### Quality assessment

The quality of included studies were assessed by two investigators independently on the basis of Newcastle-Ottawa Scale (NOS) [29], which based on three aspects: selection, comparability and exposure. Studies with a score of 5 points or higher were considered to be of high quality.

### Statistical analysis

The HWE of the genotype distributions in the controls of the include studies were tested by the Chi-square test, *P* < 0.05 was considered statistically significant. Studies with the controls not in HWE were subjected to a sensitivity analysis [30]. The power analysis was calculated by using the Power and Sample Size Program software [31]. The associations of the *HTR3A and HTR3E* polymorphisms with the risk of IBS were assessed by the pooled ORs with the corresponding 95% CIs under the following genetic models: allele model, codominant model, dominant model, and recessive model. The heterogeneity between studies was determined by the Cochrane’s Q-statistic test [32], and the inconsistency was quantified with the *I*^2^ statistic. When *I*^2^ > 50% or *P*_*Q*_ ≤ 0.1, which suggest substantial heterogeneity, a random-effects model (DerSimonian-Laird method) [33] was used; otherwise, the fixed-effects model (Mantel-Haenszel method) [28] was applied. Sensitivity analysis was conducted by sequentially omitting each study to validate the reliability of the results. Publication bias was examined with Begg’s funnel plot and Egger’s test [34], and *P* < 0.05 was considered statistically significant. All analyses were conducted using the RevMan 5.1 and STATA 12.0 software packages.

## SUPPLEMENTARY MATERIALS FIGURE AND TABLE



## Abbreviations

5-HT3: serotonin receptor type 3
IBS: Irritable bowel syndrome
IBS-D: diarrhea predominant irritable bowel syndrome
ORs: odds ratios
95% CIs: 95% confidence intervals
HWE: Hardy-Weinberg equilibrium
SNPs: single nucleotide polymorphisms
MAF: minor allele frequencies
EAS: East Asian population
AMR: Ad Mixed American population
EUR: European population
CNKI: Chinese National Knowledge Infrastructure
PRISMA: Preferred Reporting Items for Systematic Reviews and Meta-Analyses
NOS: Newcastle-Ottawa Scale
