# Supplementary material for: HTR3A and HTR3E gene polymorphisms and diarrhea predominant irritable bowel syndrome risk: evidence from a meta-analysis
Source: Oncotarget. 2017 Jul 29;8(59):100459–68. doi: 10.18632/oncotarget.19682 (PMC5725034; doi:10.18632/oncotarget.19682)
Supplement: Supplementary file 1 [file oncotarget-08-100459-s001.pdf]

HTR3A and HTR3E gene polymorphisms and diarrhea predominant irritable bowel syndrome risk: evidence from a meta-analysis

SUPPLEMENTARY MATERIALS

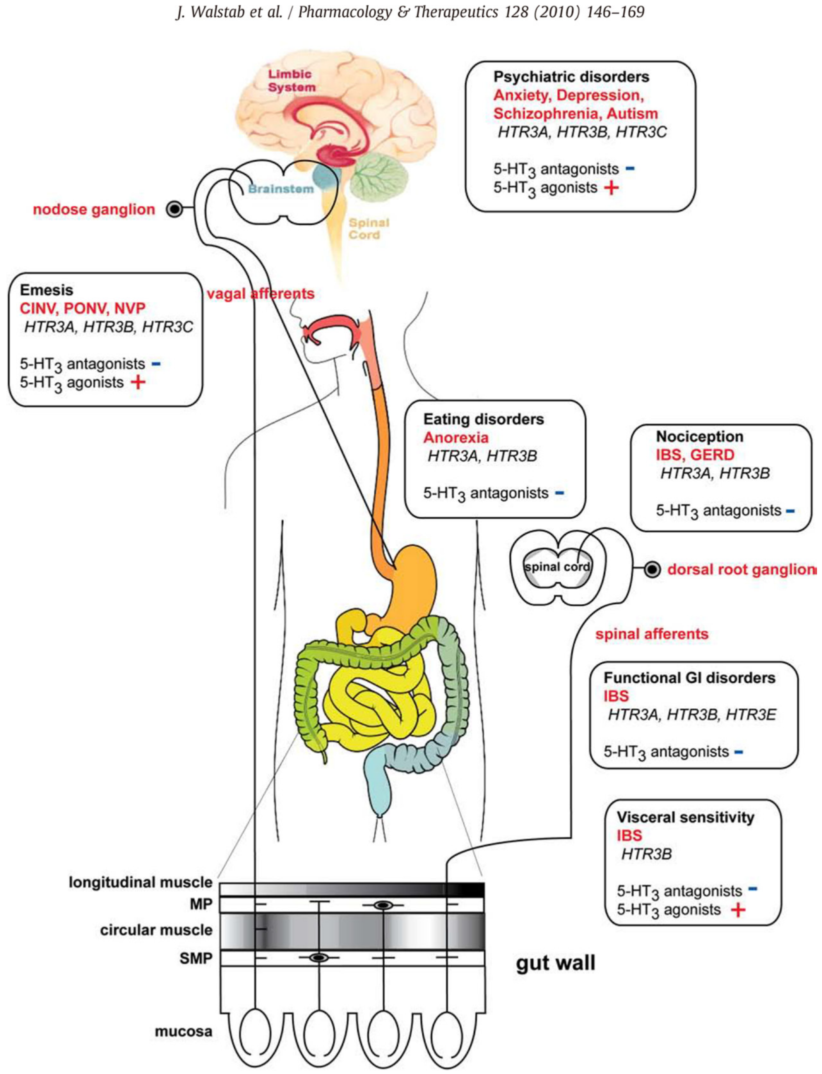

Supplementary Figure 1: The roles of 5-HT3 receptors in diseases. (+) action of 5-HT3 agonist:deteriorates symptoms by exciting the receptor, (−) action of 5-HT3 antagonist: improves symptoms by blocking the receptor, figure adapted from (Walstab, et al., 2010).

Supplementary Table 1: Quality assessment according to the Newcastle-Ottawa scale

First Author	Selection	comparability	exposure	Total scores
Gu 2015 [13]	3	2	1	6
Kapeller 2008 [11]	4	2	1	7
Kilpatrick 2011 [26]	3	2	2	7
Zhang 2013 [12]	3	2	1	6
Zhang 2016 [14]	3	2	2	7
